# Assuring Potato Tuber Quality during Storage: A Future Perspective

**DOI:** 10.3389/fpls.2017.02034

**Published:** 2017-11-28

**Authors:** M. C. Alamar, Roberta Tosetti, Sandra Landahl, Antonio Bermejo, Leon A. Terry

**Affiliations:** Plant Science Laboratory, Cranfield University, Bedfordshire, United Kingdom

**Keywords:** *Solanum tuberosum*, dormancy, sprouting, reducing sugars, phytohormones

## Abstract

Potatoes represent an important staple food crop across the planet. Yet, to maintain tuber quality and extend availability, there is a necessity to store tubers for long periods often using industrial-scale facilities. In this context, preserving potato quality is pivotal for the seed, fresh and processing sectors. The industry has always innovated and invested in improved post-harvest storage. However, the pace of technological change has and will continue to increase. For instance, more stringent legislation and changing consumer attitudes have driven renewed interest in creating alternative or complementary post-harvest treatments to traditional chemically reliant sprout suppression and disease control. Herein, the current knowledge on biochemical factors governing dormancy, the use of chlorpropham (CIPC) as well as existing and chemical alternatives, and the effects of pre- and post-harvest factors to assure potato tuber quality is reviewed. Additionally, the role of genomics as a future approach to potato quality improvement is discussed. Critically, and through a more industry targeted research, a better mechanistic understanding of how the pre-harvest environment influences tuber quality and the factors which govern dormancy transition should lead to a paradigm shift in how sustainable storage can be achieved.

## Introduction

Potato tubers (*Solanum tuberosum*) have been cultivated for more than 6000 years. Currently, potato is the fourth most important crop produced crop worldwide with an annual production of *ca.* 382 MT. Europe and Asia are the biggest producers with a share of 40.7% each, followed by America and Africa (12.6 and 4.5%, respectively) (FAOSTAT, 2014^1^). Potatoes provide an excellent source of nutrients and vitamins, but year-round availability depends on industrial-scale storage, especially in countries which rely on an annual crop. In the United Kingdom, approximately half of the total harvested tubers are stored for up to 11 months (Dale, 2014). Sub-optimal handling, poor tuber quality, and deficient post-harvest storage can lead to significant amounts of waste. The United Kingdom recorded overall losses of 17% (770,000 tons) in 2012, where premature sprouting and rotting during storage was the main cause of wastage (Terry et al., 2011; Pritchard et al., 2012). The United Kingdom outlined a strategy for a more sustained and secure food system in its Food Standard Agency (FSA) Strategic Plan 2015–2020, which aims, among several targets, to reduce waste (Food Standards Agency [FSA], 2015). This strategy is aligned with consumers’ requirements of improved nutritional value and sensory attributes, and with new regulation demanding the reduction of agrochemical usage (Lacy and Huffman, 2016).

Current challenges in the potato industry include the preservation of tuber quality throughout storage, restriction of isopropyl-*N*-(3-chlorophenyl) carbamate (chlorpropham or CIPC) residues (mainly for ware potatoes destined for processing), control of sweetening processes, and ensuring tuber marketability (visual appearance is the main factor driving consumers purchase of fresh potatoes; Terry et al., 2013).

## Factors Governing Dormancy

Dormancy break in potato tubers is a physiological phenomenon that is regulated by both exogenous (environmental factors) and endogenous signals (Sonnewald and Sonnewald, 2014). The relative concentration of several biochemical compounds such as plant growth regulators [*viz*. abscisic acid (ABA), auxins, cytokinins (CKs), gibberellins (GAs), ethylene, and strigolactones (SLs)] and other compounds (*viz.* carbohydrates and organic acids) are believed to orchestrate the onset and further development of dormancy break (Sonnewald, 2001; Viola et al., 2007; Pasare et al., 2013).

Endogenous ethylene is required at the earliest stage of dormancy initiation (endodormancy induction) (Suttle, 1998); however, its role during dormancy and sprouting is still unclear. Exogenous ethylene (10 μL L^-1^) has been reported to break endodormancy following short-term treatments (Foukaraki et al., 2014), but also to inhibit sprout growth and promote ecodormancy when supplied continuously – either starting immediately after harvest or at first indication of sprouting (Foukaraki et al., 2016a). However, work carried out on cv. Russet Burbank minitubers showed that ethylene was not involved in hormone-induced dormancy break (Suttle, 2009). These findings support the suggestion that the effect of ethylene depends on the physiological state of potato tubers.

The role of ABA is better understood. It is well known that a sustained synthesis and action of ABA is required for dormancy induction and maintenance (Suttle, 2004; Mani et al., 2014). That said, although ABA levels decrease as endodormancy weakens, there is no evidence of an ABA threshold concentration for dormancy release (Biemelt et al., 2000; Destefano-Beltrán et al., 2006; Suttle et al., 2012; Ordaz-Ortiz et al., 2015). It is also known that there is cross-talk between ABA and other phytohormones (Chang et al., 2013), as well as with sugar metabolic pathways, which facilitates the onset of dormancy break and further sprouting (Brady, 2013). Nevertheless, the increase in ABA as a result of exogenous ethylene application has been postulated to delay dormancy break (Foukaraki et al., 2016b). Concomitant to the ABA decline, there is an increase in sucrose contents, which is considered a prerequisite for bud outgrowth (Viola et al., 2007; Sonnewald and Sonnewald, 2014). In this context, auxins are essential for their role in vascular development. Auxins favor the symplastic reconnection of the apical bud region – a discrete cell domain which remains symplastically isolated throughout tuberisation. This reconnection is, therefore, essential for sucrose to reach the meristematic apical bud. High sucrose levels promote trehalose-6-phosphate accumulation (T6P) which supports sprouting probably decreasing sensitivity to ABA (Debast et al., 2011; Tsai and Gazzarrini, 2014).

It has also been demonstrated that, CKs and GAs are required for the reactivation of meristematic activity and sprout growth (Hartmann et al., 2011). Just prior to dormancy break, an increase in both cytokinin concentration and sensitivity have been reported as key factors for meristematic reactivation (Suttle, 2004). Furthermore, CKs coordinated with auxins stimulate sprout elongation (Aksenova et al., 2013). Sensitivity to GAs, which is negatively affected by SLs, increases throughout post-harvest storage and is possibly responsible for sprout vigor (Roumeliotis et al., 2012). SLs may be related to paradormancy establishment instead of eco- and endodormancy since they are key as regulators of lateral bud development (Pasare et al., 2013).

Optimum length of dormancy differs depending on cultivars and final usage of potato tubers. Thus, longer dormancy and delayed sprouting (at a desired time) would be best for ware potatoes storage, while accelerated sprouting would be preferable for seed potatoes. As reviewed by Eshel and Teper-Bamnolker (2012), sprouting has been induced in seed potatoes by the application of “Rinditie” (commercial mixture of ethylene chlorohydrin, ethylene dichloride, and carbon tetrachloride), bromoethane, carbon disulphide, and GAs (Sonnewald and Sonnewald, 2014).

## Use of Cipc During Potato Storage

Suppression of sprout growth in potato tubers represents a crucial step to manage potato quality during storage. Sprouting can be inhibited by the application of chemical sprout suppressants and by controlling environmental conditions, e.g., cold storage, tuned humidity and regulated gas composition conditions. Due to its high efficacy, CIPC is the world’s most utilized sprout suppressant chemical; it inhibits meristematic cell division, delaying sprout development. Nevertheless, concerns about CIPC usage have increased following studies which described toxic and carcinogenic properties of CIPC and its metabolites (Balaji et al., 2006; El-Awady Aml et al., 2014). However, evidence on the apparent toxicity of CIPC is sparse.

The use of CIPC is covered by the Code of Practice for using plant protection products (DEFRA, 2006). To deal with the continuous updates and concerns over exceedances in CIPC regulation, the United Kingdom assembled in 2008 the Potato Industry CIPC Stewardship Group (PICSG), which is supported by the potato industry and CIPC-related companies. From July 2017, new legislation came into force establishing that CIPC applications (36 g ton^-1^ for processing potatoes and 24 g ton^-1^ for fresh market tubers) must be done through ‘active recirculation’ of storage air by fans to optimize CIPC application (AHDB, 2017). Thus, increasing legislation constraints are driving the potato industry to seek alternative novel technologies which are able to extend post-harvest storage while maintaining tuber quality. Industry aims to provide high quality potatoes with contained costs for storage management; for this reason, it is pivotal to have sprout suppression technologies which can be exploited in the long-term.

## Sprout Control During Post-Harvest – Physical and Chemical Alternatives to Cipc

Premature sprouting is one of the major causes of loss during post-harvest storage of ware potatoes, since it reduces the number of marketable tubers and fresh weight due to water loss from sprout surfaces, and the remobilisation of starch (Sonnewald and Sonnewald, 2014). The sprout suppressant CIPC is, in general, commercially applied as a thermal hotfog (single or multiple treatments) during prolonged potato storage (Blenkinsop et al., 2002). However, legislative bodies are constraining its use. Alternatives (or supplements) to traditional sprout control include hydrogen peroxide plus (HPP) (Al-Mughrabi, 2010; Mani et al., 2014), 1,4-dimethylnaphthalene (1,4-DMN) (de Weerd et al., 2010), UV-C (Cools et al., 2014), essential oils and ethylene. Continuous exogenous ethylene supplementation has been commercially approved as a sprout suppressant in United Kingdom by the Chemicals Regulation Directorate (CRD) (Briddon, 2006); yet, the way in which ethylene inhibits sprout growth has not been completely clarified. It is known that ethylene supplementation can increase the content of reducing sugars in tubers (Daniels-Lake et al., 2005), which negatively affects processed potato quality. Nevertheless, late ethylene supplementation (at eye movement stage) was efficacious at delaying tuber sprouting, and more effective preventing accumulation of reducing sugars when compared to early supplementation (applied after curing and from the beginning of storage) (Foukaraki et al., 2014). Therefore, late ethylene supplementation may reduce storage costs whilst providing high quality tubers. The ethylene-induced increase in ABA levels may explain this delay of dormancy break (Foukaraki et al., 2016b).

Low temperature conditions is a worldwide used storage technology, which delays tuber sprouting. Besides low temperature, other physical methods such as gamma radiation have been shown to be effective in controlling sprout growth (Rezaee et al., 2013); yet its use is subject to strict legislation. Short wave ultraviolet radiation has been likewise suggested as an alternative or complementary method for sprout control (Pristijono et al., 2016). Thus, moderate UV-C doses (5–20 kJ m^-2^) have been found to suppress sprout length and sprout incidence in a range of potato cultivars when applied at first indication of sprouting (Cools et al., 2014). The direct deleterious effect on the meristematic tissue, combined with potential changes in tuber biochemistry have been postulated as mechanisms by which gamma and UV-C radiation control sprouting.

The use of alternative chemical sprout suppressants during post-harvest aim at damaging the meristematic tissue to cease or disrupt cell proliferation; for example, local necrosis of the bud meristem was found after the application of mint essential oils (Teper-Bamnolker et al., 2010). Previously, Eshel et al. (2008) had shown the potential of mint oil vapor to be used at large scale to control sprouting in four commercial potatoes cultivars. Repeated applications of Talent^®^, trade name for a monoterpene (carvone) derived from caraway seed, can inhibit sprout growth for up to a year; however, the number of applications required may make this solution uncompetitive when compared to CIPC (Npcs Board of Consultants and Engineers, 2007). Additionally, both, HPP and 1,4-DMN have been successfully applied as a fog to control sprouting. Afek et al. (2000) achieved complete sprout suppression (6 months at 10 ± 1°C) when potatoes were treated with HPP for 10 h; whereas the use of 1,4-DMN (at a rate of 20 μL L^-1^) needs more investigation to elucidate whether it is environmentally safe (Oteef, 2008).

## Effect of Pre-Harvest Factors and Storage Conditions on Tuber Quality

The quality of potatoes is established in the field and can only be preserved during post-harvest. Abiotic factors influencing tuber maturity, cultivar- and season-variability have great impact on final quality. Driskill et al. (2007) reported that the processing quality (fry color) of younger tubers (late planting) was better than that of tubers planted earlier. High nutrient demand on soil for good tuber quality requires high organic matter and nitrogen input (Nesbitt and Adl, 2014). Sustainable agricultural practices such as balanced fertilizer regimes improved not only tuber yield but also marketing quality of potato (e.g., tuber size; Tan et al., 2016). Vine desiccation (diquat, comm. Reglone^®^) is another factor which strongly impacts quality; it triggers both maturation of the tuber periderm and stolon release, and in seed potato production it can also control tuber size. To manage all these variables a multifactorial approach is recommended to mitigate side effects which may affect quality (De Meulenaer et al., 2008).

After harvest, tuber quality management aims to delay dormancy break and limit weight loss and sweetening of potatoes. Senescent sweetening is a natural process that occurs as a result of tuber aging; it is irreversible and involves cellular breakdown. Following cellular breakdown, structural and non-structural carbohydrates are depolymerized by hydrolytic enzymes. To delay this process, correct storage conditions are crucial. Cold storage is commonly used to control sprouting, yet temperature management depends on the intended market: tubers for the fresh market can be stored at temperatures below 7°C while tubers destined for the processing market need higher temperature (8–13°C) to preserve frying quality. Quality loss is also caused by ‘cold-induced sweetening’ when sucrose hydrolysis leads to reducing sugars accumulation; although it can be partially reversed by temperature reconditioning (Driskill et al., 2007). Cold-induced sweetening, however, does not only depend on post-harvest storage conditions but also on potato variety (Elmore et al., 2016) and growing location (Muttucumaru et al., 2017). Low levels of reducing sugars are preferred in processing potatoes since when tubers are cooked at high temperatures (>120 °C) the Maillard reaction can occur. During the Maillard reaction, reducing sugars are responsible for the browning (non-enzymatic reactions) of the product (French fries, crisps) and, as a side effect acrylamide may also accumulate. As recently reviewed by Muttucumaru et al. (2017), the principal pathway for acrylamide formation is the deamination and decarboxylation of free asparagine under high temperatures and its reaction with reducing sugars. Potato is one of the major contributors to dietary acrylamide (Group 2A, ‘probably carcinogenic to humans’) intake in the European Union (Borda and Alexe, 2011). The European Commission issued ‘indicative’ levels (not regulatory or safety thresholds) of acrylamide in food in 2011, which were revised downward for many products in 2013 (e.g., crisps = 100 μg kg^-1^ and French fries = 600 μg kg^-1^). In this context, FoodDrinkEurope (2013) created a ‘Toolbox’ which compiles different strategies from the food industry to reduce acrylamide formation by modifying food processing.

As previously mentioned, continuous ethylene supplementation is currently used as a sprout suppressant during storage; yet, it can induce sucrose hydrolysis (ethylene-induced sweetening). A recent study has shown that this type of sweetening can be prevented with a single application (24 h) of 1-methylcyclopropene (1-MCP) prior to early and late ethylene supplementation (Foukaraki et al., 2016a). The impact of CO_2_, another storage extension gas, on frying quality is less clear. Studies on processing potato varieties showed negative effects on fry color when ethylene and CO_2_ were applied together. Despite this, cultivar, gas concentration and timing, and seasonality strongly affect responses of tubers to CO_2_ treatment (Daniels-Lake, 2013).

## The Role of Genomics in Potato Quality Improvement

Breeding programs aim to develop new cultivars with improved features (productivity, pathogens- and stress-resistance). Due to the complex genetic heterogeneity of modern potato cultivars, conventional potato breeding requires approximately 10 years for the phenotypic selection cycle: from crossing to variety release (Slater et al., 2014). The increasing knowledge about the geno-phenotypic relationships and the availability of new technologies has allowed for the development of ‘precision breeding.’ Precision breeding increases the efficiency in selection of targeted traits through genetics techniques (e.g., marker assisted selection, MAS) and shorten the selection cycle (Gebhardt, 2013). These have been used to identify disease resistance genes in wild relatives and to cross them into commercial potatoes (Gebhardt et al., 2014). The knockout of the vacuolar invertase gene, *Vinv*, in potato tubers resulted in cold induced-sweetening inhibition and high quality processed tubers (Clasen et al., 2016). Other simple traits have been identified for tuber shape, tissue colors, and eye depth (Slater et al., 2014). Despite these advances, the majority of commercially interesting traits are complex. Post-harvest traits such as tuber yield, starch content, crisp color, or bruising susceptibility are regulated by multiple genetic and environmental factors (Gebhardt, 2013). The complexity of these traits requires a deeper knowledge of geno-phenotypic interactions and more powerful technologies.

The release of the potato genome (Potato Genome Sequencing Consortium, 2011), improved gene annotations and linkage maps led to the development of new genetic resources (e.g., single nucleotide polymorphisms arrays, SNPs arrays; genome-wide association studies, GWAS). These new technologies have been exploited to analyze the regulation of complex traits and QTL (quantitative traits loci). The Illumina Infinium 8303 SNPs Potato Array (Hamilton et al., 2011; Douches et al., 2014) allowed improved understanding of genetic control for several complex traits such as tuber dormancy and starch metabolism (Schreiber et al., 2014; Sharma et al., 2014). GWAS was proved beneficial when clarifying other quality traits of potato tubers [i.e., maturity and ‘after baking darkening’ (Björn et al., 2008; Ramakrishnan et al., 2015)]. Precision breeding has decreased breeding costs, reducing field-related expenses, and simplified the selection of interesting relatives. Furthermore, continuous progress of new genetic technologies has allowed the costs of genotyping to be cut, and the quicker screening of large populations to be more accessible. Other ‘omics’ technologies, such as transcriptomics, proteomics, and metabolomics, can be coupled with genomics and may improve the identification of quality candidate traits (Gebhardt, 2013).

Gene editing technologies are greatly increasing due to the generation of transgenic-free genetically modified organisms; additionally, gene editing allows targeted mutation with high specificity and precision in selected loci (Georges and Ray, 2017). This is achieved by inducing breaks in the genome and utilizing DNA repair pathways to modify target genes (Curtin et al., 2012). Double-strands breaks (DBS) are induced by endonuclease enzymes (like transcription activator-like effector nucleases (TALEN), and CRISPR-associated (Cas) endonucleases). Following DBS, the DNA repair pathways may inactivate a gene (knockout) through a non-homologous end joining (NHEJ) pathway, or replace-insert a gene through homologous recombination (HR) pathway (Symington and Gautier, 2011). Gene editing with insertion is often achieved by combining the action of *Agrobacterium tumefaciens* with plant viruses. This combination showed very promising results when *Agrobacterium* was coupled with DNA virus *Geminivirus* replicon (GVR), which allows a larger carrying capacity compared to RNA viruses. Potato plants modified with this technique exhibited reduced herbicide susceptibility (Butler et al., 2016).

## Conclusion

In order to assure future potato tuber quality, industry and academic communities have to work together while considering consumers preferences. Deploying molecular and improved phenotyping techniques to increase the knowledge of mechanisms which mediate physiological responses during pre-harvest production, post-harvest storage and processing (*viz.* acrylamide formation) will improve tuber quality. These combined efforts will benefit the development of new cultivars with improved features and provide guidelines for more sustainable agricultural techniques and storage strategies. At the same time, alternative pre- and post-harvest technologies have to be embraced and further implemented by the potato industry. Through a more industry targeted research, the combination of genomics, pre- and post-harvest technologies will aid the preservation, enhancement, and viability of future tuber quality.

## Author Contributions

MA: Paper writing (35%). RT: Paper writing (35%). SL: Paper writing (10%). AB: paper writing (10%). LT: Overall supervision (10%).

## Conflict of Interest Statement

The authors declare that the research was conducted in the absence of any commercial or financial relationships that could be construed as a potential conflict of interest.

## Footnotes

1 http://www.fao.org/faostat/
